# Impression material accuracy for palatal orthodontic miniscrews

**DOI:** 10.1007/s00056-020-00245-3

**Published:** 2020-09-08

**Authors:** Natalie Schenz, Vincent Schwarz, Romed Hörmann, Adriano G. Crismani

**Affiliations:** 1 University Hospital for Orthodontics, Medical University of Innsbruck, Anichstraße 35, 6020 Innsbruck, Austria; 2 Department of Cranio-Maxillofacial Surgery, Faculty of Medicine of the Kepler University Linz, Krankenhausstraße 9, 4020 Linz, Austria; 3 Division of Functional and Clinical Anatomy, Medical University of Innsbruck, Müllerstraße 59, 6020 Innsbruck, Austria

**Keywords:** Polyvinylsiloxane, Alginate, Polyether, Temporary anchorage devices, Orthodontic anchorage procedures, Polyvinylsiloxan, Alginat, Polyether, Vorläufige Verankerungsvorrichtungen, Kieferorthopädische Verankerungsverfahren

## Abstract

**Purpose:**

This study investigates the accuracy of abutment transfer with current impression materials and provides a concise overview, including other relevant factors, in order to enable clinicians to make an informed decision about the optimal impression for this treatment procedure.

**Methods:**

In all, 96 impressions of a cadaver head with two orthodontic miniscrews in place were taken with four common impression materials by two observers and using two methods of application. After pouring with a standard type IV stone and abutment transfer, all models and the upper jaw (which had been separated from the head) were scanned in a standard model scanner (Zirkonzahn® [Zirkohnzahn GmbH, Gais, Italy] S600 ARTI) and evaluated using a computer-aided design (CAD) program (GOM-Inspect [Gesellschaft für optische Messtechnik m.b.H., Braunschweig, Germany]). The deviations were measured at six points per screw and statistically evaluated with SPSS® (IBM, Chicago, IL, USA).

**Results:**

Optimal values were obtained with biphasic polyvinylsiloxane, while monophasic polyvinylsiloxane, alginate and polyether also resulted in acceptable accuracy. Observer experience showed no effect and the method of application had only a minor effect on accuracy.

**Conclusions:**

Within the limitations of this study, it seems that all impression materials are suitable for miniscrew abutment transfer, provided that methods of intraoral adaptation of the orthodontic appliance can be employed. If higher accuracy is needed or for clinicians with less experienced, a biphasic polyvinylsiloxane impression with the putty-wash technique should be used as this combination reduces setting time. The most cost-effective version, alginate, can be used if the consequences of greater deviations can be handled. Caution is advised with polyether if undercuts are present.

## Abstract (de)

**Zielsetzung:**

Die vorliegende Studie untersucht die Genauigkeit von Abformmaterialien für den Abutment-Transfer und versucht, einen kompakten Überblick über relevante Faktoren zu geben, um eine fundierte Entscheidung für die optimale Abformtechnik bei diesem Behandlungsverfahren zu ermöglichen.

**Material und Methoden:**

Insgesamt 96 Abdrücke am Oberkiefer eines Körperspenders mit 2 inserierten kieferorthopädischen Minischrauben wurden von 2 Untersuchern mit 4 gängigen Abformmaterialien unter Verwendung von 2 Applikationsmethoden durchgeführt. Nach dem Ausgießen mit Typ-IV-Superhartgips und Abutment-Transfer wurden alle Modelle und der vom Kopf getrennte Oberkiefer in einem Modellscanner gescannt (Zirkonzahn® [Zirkohnzahn GmbH, Gais, Italien] S600 ARTI) und in einem CAD(„computer-aided design“)-Programm (GOM-Inspect [Gesellschaft für optische Messtechnik m.b.H., Braunschweig, Deutschland]) verglichen. Die Abweichungen wurden an 6 Punkten pro Schraube gemessen und mit SPSS® (IBM, Chicago/IL, USA) statistisch ausgewertet.

**Ergebnisse:**

Optimale Ergebnisse wurden mit dem biphasischem Polyvinylsiloxan erzielt, während das monophasische Polyvinylsiloxan, Alginat und Polyether ebenfalls zu einer akzeptablen Genauigkeit führten. Die Erfahrung des Untersuchers zeigte keinen Effekt, und die Applikationsmethode hatte nur einen geringen Einfluss auf die Abformgenauigkeit.

**Schlussfolgerung:**

Im Rahmen dieser Studie scheinen alle Abformmaterialien für den Abutment-Transfer von Minischrauben geeignet zu sein, vorausgesetzt es können Methoden zur intraoralen Anpassung der kieferorthopädischen Apparatur vorgenommen werden. Ist eine höhere Genauigkeit notwendig oder der Untersucher unerfahren, sollte zur Abformung ein biphasisches Polyvinylsiloxan in der Doppelmischabformung verwendet werden, da diese Kombination die Abbindezeit verkürzt. Die kosteneffizienteste Version, Alginat, kann verwendet werden, wenn der Behandler mit den Folgen größerer Abweichungen umgehen kann. Sind Unterschnitte vorhanden, so ist besondere Vorsicht bei der Verwendung von Polyether empfohlen.

## Introduction

In recent years, orthodontic miniscrews have been increasingly used in orthodontics as an integral part of modern therapeutic approaches [4, 16]. They are the most commonly applied skeletal anchorage system (temporary anchorage devices, TADs) [17].

While palatal orthodontic implants focus on osseointegration to achieve stability, the obvious advantage of orthodontic miniscrews lies in the use of primary stability resulting from mechanical retention, which also makes them far easier to remove [7, 14, 16]. Screw systems with and without abutments [26, 27] as well as abutment-carrying orthodontic implants [7] are available for palatal cortical anchorage. In the present study, an abutment-carrying orthodontic miniscrew system, based on the OrthoEasy® Pal system (OrthoEasy® Pal, Forestadent Bernhard Förster GmbH, Pforzheim, Germany), was designed for palatal paramedian insertion, which enables both molar distalization and rapid maxillary expansion (RME) to be carried out [26, 27]. An important factor in the use of this palatal cortical anchorage is attributable to a correct transfer of the appliance position. A few approaches have been described, such as the use of individualized impression abutments [27], orthodontic implants with impression abutments [7], adaptation of the orthodontic mechanism or [8] the abutment-carrying palatal miniscrew, as a means of avoiding potential problems during transfer.

The impression material used plays a key role in respect of detail accuracy, which is dependent on various factors such as the wettability, elastic properties and dimensional stability of the material. A distinction also needs to be made between customized and prefabricated impression trays with their particular advantages and disadvantages.

Recent studies investigating the suitability of alginate and polyether as impression materials for orthodontic use established that polyether has greater accuracy than alginate, although both impression materials proved to be accurate enough for orthodontic use. Whereas the impression tray design was found to have an influence when polyether was used as the impression material, it had no such effect in the case of alginate impressions [23].

Since Möhrmann et al. started development of the first CAD-CAM (computer-aided design and manufacturing) system in dentistry, three-dimensional (3D) systems have rapidly advanced [20]. Today 3D imaging has become indispensable as an integral part of all dental specialisms, with scanning of dental casts and intraoral scanning delivering particularly promising results [5].

Vogel et al. [25] studied the completeness of impression scans as a function of malocclusion and concluded that the accuracy of model scans currently exceeds the quality of impression scans. A study by Dalstra et al. [9] revealed that the virtual measurement of digital orthodontic models permits less variability than the corresponding manual measurement of casts.

The aim of this study was to evaluate the 3D accuracy of impression materials commonly in use for transfer of the abutment position of an orthodontic miniscrew system (OrthoEasy® Pal, Forestadent Bernhard Förster GmbH, Pforzheim, Germany). In some studies, alginates are not recommended for taking impressions of miniscrews because of their tendency of tearing compared with elastomeric impression materials [24]. However, the authors are not aware of any studies published to date regarding this particular use.

## Materials and methods

A total of 96 impressions were prepared from the maxilla of a body donor (female, 90 years) with two orthodontic miniscrews (OrthoEasy® Pal palatal pin, 8 × 1.7 mm, Forestadent Bernhard Förster GmbH, Pforzheim, Germany) which were inserted in a 2 mm paramedian position on a connecting line between the first premolars. To ensure comparability, perforated maxillary impression trays (SUP B0‑4, Martin® Elite, Gebrüder Martin GmbH & Co. KG, Mühlheim, Germany) were used for all the impressions with the appropriate adhesive for alginate or polyether. The alginate was mixed using a standard alginate mixing device (Migma 200 alginate mixer, Mikrona, Spreitenbach, Switzerland) according to the water/powder ratio recommended by the manufacturer. A universal mixing device (Pentamix™ light, 3M Austria GmbH, Vienna, Austria) was available for all the other impression materials. All four of the impression materials used were provided by 3M Espe Dental Products (3M Austria GmbH, Vienna, Austria). First, a standard alginate (Palgat™, 3M Austria GmbH, Vienna, Austria) was selected. A monophasic polyvinylsiloxane (PVS, Imprint™ 4 Preliminary, 3M Austria GmbH, Vienna, Austria) served as another impression material; this is specified as an alternative to alginate because of its fast setting time (2 min). A biphasic polyvinylsiloxane (Imprint™ 4 Penta™ Putty and Imprint™ 4 light, 3M Austria GmbH, Vienna, Austria) with high accuracy was also used. The fourth impression material selected was a polyether (Impregum™ Penta™, 3M Austria GmbH, Vienna, Austria), which is regarded as a standard for implant impression-taking and deemed highly accurate and dimensionally stable [13].

The body donated to the Division of Clinical and Functional Anatomy of the Medical University had given their informed consent for its use for scientific and educational purposes prior to death [19]. It was preserved using an arterial injection of a formaldehyde–phenol solution/an alcohol–glycerin solution and immersion in phenolic acid in water for 1–3 months [21].

**Fig. 1:**
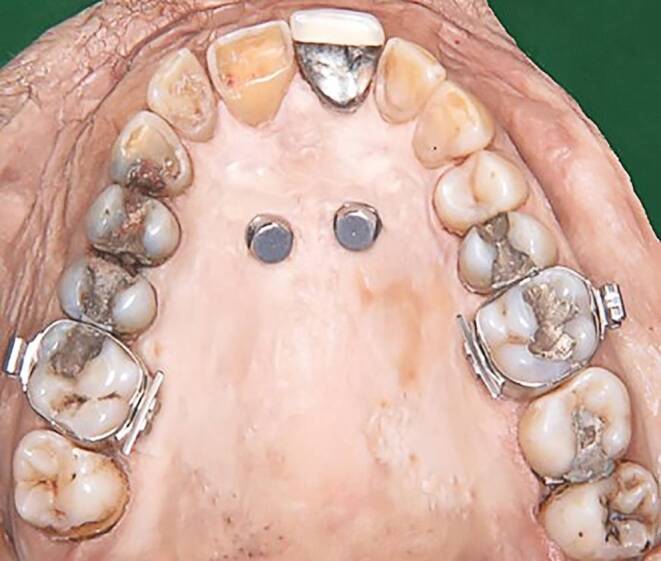
Upper jaw with two orthodontic miniscrews and impression caps, bands on first molars Oberkiefer mit 2 kieferorthopädischen Minischrauben und Abformkappen, Bändern an den ersten Molaren

After placement of the impression caps on the palatal miniscrews (Fig. 1) and selection of the right size (L) of impression tray, impressions were taken in the following sequence: PVS preliminary, PVS biphasic, alginate, polyether.

The impressions were taken by two observers (experienced, inexperienced) with six impressions per material being taken. This number of impressions per subgroup was chosen because the analysis of variance (one-way analysis of variance [ANOVA]) suggested that four to eight is the optimal number per group (required power [%] = 80) [12]. Two application methods were tested for each impression material (in a perforated metal tray with and without local application). While alginate was solely applied to the impression tray in one group, in the other group it was additionally applied manually to the dental arch and the peri-implant region. An impression syringe provided by the manufacturer (Penta Elastomer Syringe, 3M Austria GmbH, Vienna, Austria) was also used for the impressions created with polyether and PVS preliminary. In the case of biphasic PVS, the putty-body component was applied to the impression tray and the light-body component was applied locally around the teeth and the abutments of the miniscrews, as a putty-wash combination. The second method involved corrective impression-taking with the putty component in the impression tray and a special separating foil (Plicafol, GS Folienfertigung, Lebach, Germany), which created space for the light component that was applied in a separate step. All the impressions were executed according to manufacturers’ instructions and promptly poured. Before pouring, the laboratory analogues for the palatal miniscrews were positioned in the impression caps and fit was checked.

The alginate impressions were poured immediately, and the impression trays were removed as soon as the temperature of the plaster had returned to normal. By contrast, all the other materials were not poured until a period of 24 h had elapsed. An ISO type 4 stone (SILKY-ROCK yellow, Whip Mix, Louisville, KY, USA) was used for pouring. It was activated with water at a ratio of 23 ml water per 100 g plaster powder, mixed for 60 s in a vacuum mixing device (Twister, Renfert GmbH, Hilzingen, Germany) and then applied in the impressions using a vibrator (Power Vibrator KV 36, Wassermann Dental Maschinen GmbH, Hamburg, Germany). The casts were labelled with randomized numbers before scanning and evaluation.

After abutment transfer and subsequent application of a scan spray (Zirko Scanspray, Zirkonzahn GmbH, Gais, Italy) to the palatal abutments, all the models and the upper jaw (which had been separated from the head) were scanned in a model scanner (Zirkonzahn® S600 ARTI, Zirkohnzahn GmbH, Gais, Italy).

**Fig. 2:**
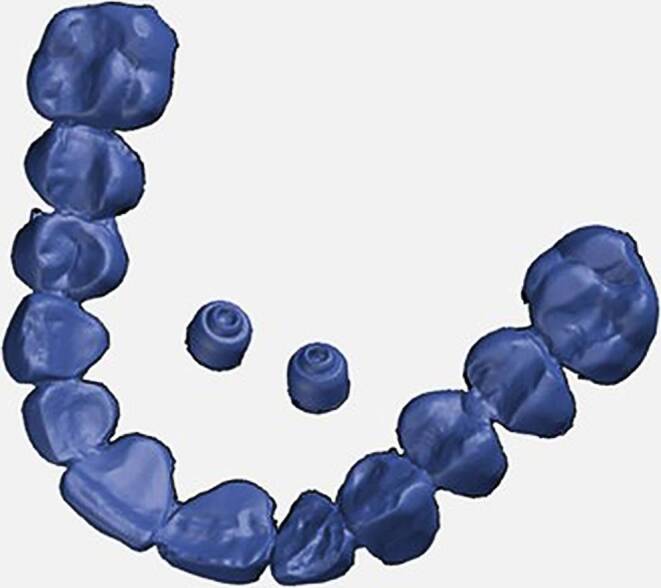
Master model Mastermodell

All the files were saved in STL format and compared in a CAD (computer-aided design) program, namely GOM Inspect version 7.5 SR1 (Gesellschaft für optische Messtechnik m.b.H., Braunschweig, Germany). So that a comparison could be made, the scan of the maxilla was loaded into the GOM Inspect interface as a “CAD body”, the dataset on the dental arch with the first molars and the palatal abutments being reduced and stored as the master model (Fig. 2).

**Fig. 3:**
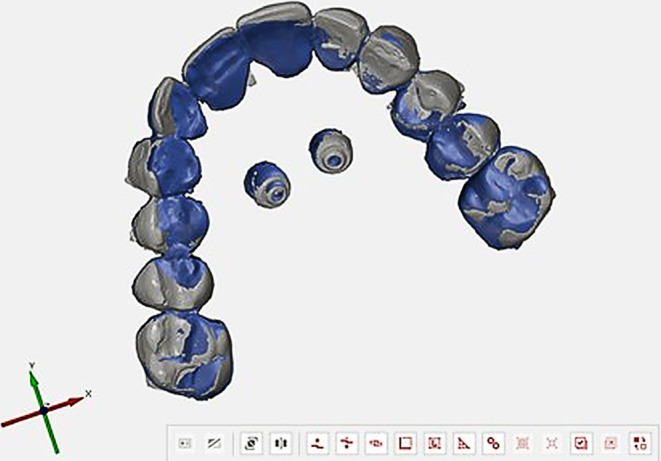
Completed pre-alignment with a first best fit Abgeschlossene Vorausrichtung mit erster optimaler Überlagerung

Each individual model scan was then loaded into the project as a grid structure and suitably trimmed. The next step was a pre-alignment of the datasets, which was mandatory for further analysis by means of CAD comparison. For pre-alignment before superimposition, STL datasets in this program are defined as body and mesh structures. Therefore, the cadaver scan was defined as the body structure and the cast scans were defined as mesh structures. Thereafter, virtual selections of the tooth-representing grid structures of the upper jaw and the model scan were performed to serve as reference in the best-fit alignment function by the GOM Inspect program (Fig. 3).

**Fig. 4:**
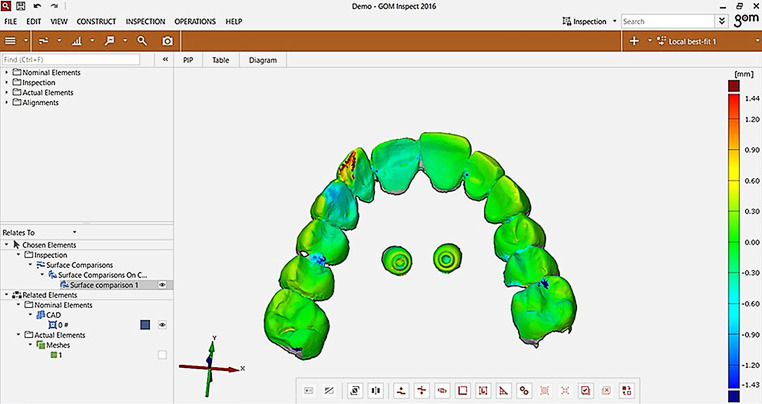
Re-orientation of the model for computer-aided design (CAD) comparison Reorientierung des Modells für den CAD(„computer-aided design“)-Vergleich

**Fig. 5:**
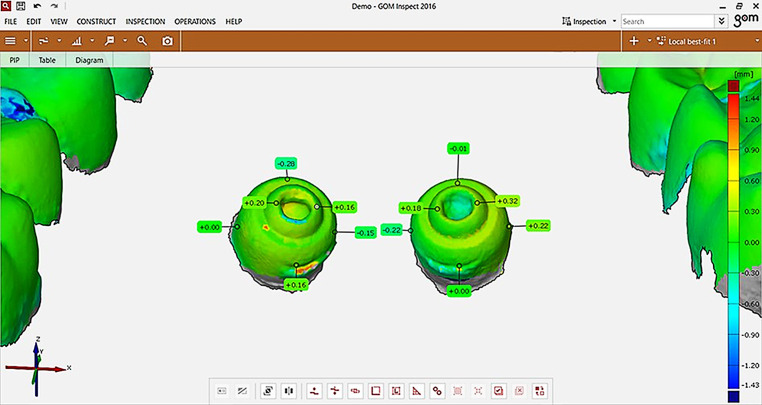
Labelling of deviations for later matching to their corresponding variables of observer, method, and material Beschriftung von Abweichungen für die spätere Zuordnung zu den entsprechenden Variablen von Untersucher, Methode und Material

After the tooth-referring local best fit, a CAD comparison was carried out and the model was reoriented in a standardized position (Fig. 4) in which 12 points were defined on the upper surface of the screws (six points per screw), eight of which were located at the abutment neck, to calculate differences in the vertical, horizontal, and transversal directions (Fig. 5). Thus, the statistical significance of the deviations could be evaluated.

Finally, the resulting values were matched to their appropriate models in order to evaluate the influence of observer, method of impression-taking, and impression material.

## Statistical analysis

A total of 1152 values were measured with 12 points per model being measured on six models per subgroup.

The means and standard deviations (SD) were calculated for each subgroup and spatial direction. The overall deviations were also ascertained. Box plot diagrams, showing the means as a thick horizontal line, were used to illustrate the results. The box is limited by an upper and lower quartile, namely a 75% and a 25% quartile. The 10% and 90% quartiles are depicted as thin horizontal lines, while a connection to the box exists via vertical lines. Any extreme values present are shown as stars or dots [15].

The measured values were given as mean ± SD unless otherwise indicated. The Mann–Whitney U test or Kruskal–Wallis test were used for statistical analysis of the nonparametric variables. Due to excessive standard deviations, the conducted univariate ANOVA (analysis of variance) with Bonferroni correction was not valid, but a test of between-subject effects was still regarded. Statistical analysis was performed with SPSS® version 23(IBM, Chicago, IL, USA). A *p*-value of less than 0.05 was considered to be statistically significant.

## Results

The results showed differences with regard to the level of deviation in the three spatial dimensions (and overall) when applying the four different materials, which were each applied by two observers using two different methods.

The values for the direction vectors can be described as follows: negative values mean that the screw on the cast model is shifted further downwards in the axial direction, further to the left in the transverse axis and further backwards in the sagittal axis compared with the original; positive values indicate the exact opposite. This explanation obviously cannot be used for the overall assessment of all directions, which is why all the values were standardized as positive for this purpose.

### General differences in observer, material, and method

#### Observer

**Fig. 6:**
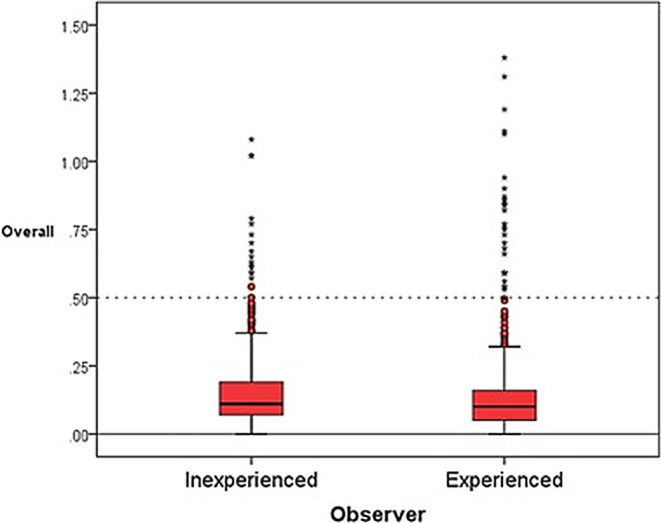
Boxplots for overall observer differences Boxplots für die Unterschiede der Untersucher insgesamt

The differences in relation to the observer showed mean values for the experienced observer which deviated less from nil than was the case for the inexperienced observer, and these differences can be regarded as significant for the transverse and sagittal direction. On the other hand, the standard deviations for the inexperienced observer were smaller in all the separate directions and overall (Table 1; Fig. 6).

**Table 1:** Observer-specific differences over all spatial planes Beobachterspezifische Unterschiede in allen räumlichen Ebenen

	Observer	*N*	Mean (mm)	SD (mm)	*p*-values
Axial	Inexperienced	192	0.037	0.250	0.425 (n. s.)
	Experienced	192	−0.006	0.284	
Transversal	Inexperienced	192	−0.053	0.122	0.003**
	Experienced	192	−0.013	0.141	
Sagittal	Inexperienced	192	−0.133	0.172	0.020*
	Experienced	192	−0.113	0.213	
Overall	Inexperienced	576	0.150	0.142	0.002**
	Experienced	576	0.144	0.179	

*N* number, *SD* standard deviation, *n.s*. not significant

*p< 0,05, **p< 0,01

#### Material

**Fig. 7:**
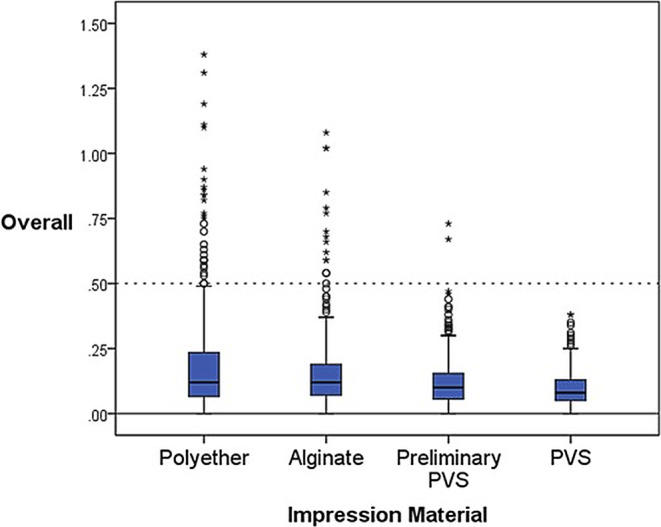
Boxplots for overall differences in materials Boxplots für die Unterschiede der Materialien insgesamt

Table 2 shows small deviations of the mean values in the sagittal and axial directions for the biphasic PVS. The significance was very high (***) in the sagittal direction and high (**) in the axial direction. Considering the transverse direction, polyether showed the smallest mean in terms of deviation. This was followed by preliminary PVS, alginate, and lastly biphasic PVS with ascending mean values. Conversely, biphasic PVS showed the lowest deviation values overall and the lowest standard deviation, followed by preliminary PVS, alginate, and polyether (Table 2; Fig. 7).

**Table 2:** Overall deviation values for materials Abweichungswerte für die Materialien insgesamt

	Impression Material	*N*	Mean (mm)	±SD (mm)	*p*-values
Axial	Polyether	96	−0.037	±0.390	0.005 (**)
	Preliminary PVS	96	0.065	±0.176	
	Alginate	96	0.040	±0.284	
	Polyvinylsiloxane	96	−0.006	±0.138	
Transversal	Polyether	96	−0.003	±0.198	0.011 (*)
	Preliminary PVS	96	−0.027	±0.099	
	Alginate	96	−0.039	±0.114	
	Polyvinylsiloxane	96	−0.064	±0.087	
Sagittal	Polyether	96	−0.163	±0.270	0.000 (***)
	Preliminary PVS	96	−0.130	±0.113	
	Alginate	96	−0.175	±0.193	
	Polyvinylsiloxane	96	−0.024	±0.118	
Overall	Polyether	288	0.205	±0.233	0.000 (***)
	Preliminary PVS	288	0.121	±0.102	
	Alginate	288	0.163	±0.167	
	Polyvinylsiloxane	288	0.098	±0.073	

*N* number, *SD* standard deviation, *PVS* polyvinylsiloxane

*p< 0,05, **p< 0,01, ***p< 0,001

#### Application method

**Fig. 8:**
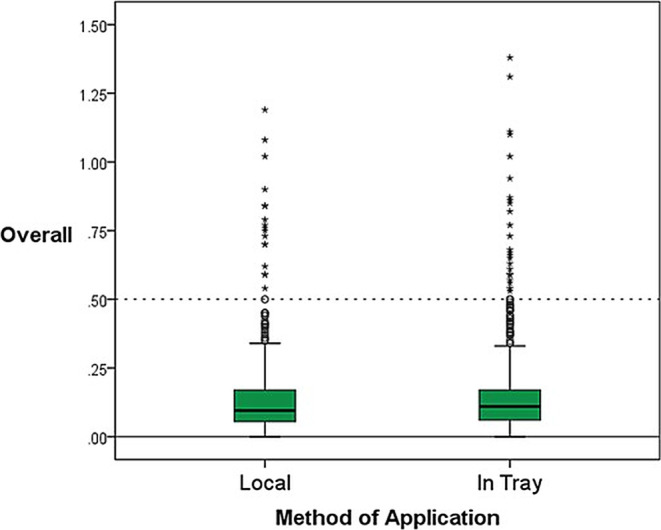
Boxplots for overall differences in method of application Boxplots für die Unterschiede der Applikationsmethode insgesamt

Statistical analysis of the influence of the method of application on abutment position revealed higher mean values for local application in the axial and sagittal direction, whereas application solely to the impression tray showed higher means in the transverse direction. However, these differences were not significant. When evaluating all the spatial dimensions, the local application method had lower means and standard deviations, which can be viewed as slightly significant with a *p*-value of 0.041 (Table 3; Fig. 8).

**Table 3:** Deviation values for different methods of application (local = additional application in a syringe or, in case of alginate, manually) Abweichungswerte für die unterschiedlichen Applikationsmethoden (lokal = zusätzliche Applikation in einer Spritze oder, im Fall von Alginat, manuell)

	Method of Application	*N*	Mean (mm)	±SD (mm)	*p*-values
Axial	Local	192	0.025	±0.220	0.898 (n. s.)
	In Tray	192	0.006	±0.308	
Transversal	Local	192	−0.020	±0.130	0.100 (n. s.)
	In Tray	192	−0.046	±0.136	
Sagittal	Local	192	−0.130	±0.198	0.346 (n. s.)
	In Tray	192	−0.116	±0.190	
Overall	Local	576	0.137	±0.148	0.041 (*)
	In Tray	576	0.157	±0.174	

*N* number, *SD* standard deviation, *n.s*. not significant

*p< 0,05

### Differences in material for each observer

#### Inexperienced observer

Table 4 shows the lowest deviation values and standard deviations in the axial direction for the inexperienced observer with the biphasic PVS. Alginate also showed low values in this direction, while at the same time—in common with polyether and preliminary PVS—it exhibited extremes spreading beyond the 0.5 mm confidence interval. The statistical significance in this case could be regarded as high (**). In the transverse direction, all the values appeared to be comparably low. These differences were not significant. Biphasic PVS showed the highest values. Viewing the sagittal direction revealed that polyether and biphasic PVS, on the one hand, achieved low values of deviation, whereas alginate reached the highest values. The statistical significance of these differences was very high (***).

**Table 4:** Deviation values for materials by inexperienced observer in all spatial directions Abweichungswerte für die Materialien bezogen auf den unerfahrenen Beobachter in allen räumlichen Ebenen

Orientation	Impression material	*N*	Mean (mm)	±SD (mm)	*p*-values
Axial	Polyether	48	0.091	±0.260	0.005 (**)
	Preliminary PVS	48	0.113	±0.205	
	Alginate	48	−0.033	±0.323	
	Polyvinylsiloxane	48	−0.024	±0.151	
Transversal	Polyether	48	−0.047	±0.150	0.806 (n. s.)
	Preliminary PVS	48	−0.048	±0.107	
	Alginate	48	−0.050	±0.132	
	Polyvinylsiloxane	48	−0.068	±0.094	
Sagittal	Polyether	48	−0.084	±0.157	0.000 (***)
	Preliminary PVS	48	−0.137	±0.113	
	Alginate	48	−0.223	±0.237	
	Polyvinylsiloxane	48	−0.090	±0.120	
Overall	Polyether	144	0.156	±0.138	0.001 (**)
	Preliminary PVS	144	0.141	±0.115	
	Alginate	144	0.192	±0.197	
	Polyvinylsiloxane	144	0.111	±0.084	

*N* number, *SD* standard deviation, *n.s*. not significant, *PVS* polyvinylsiloxane

**p< 0,01, ***p< 0,001

Polyether and preliminary PVS displayed the lowest mean values of deviation for all directions in the sum. However, biphasic PVS and preliminary PVS were excessive in terms of standard deviation (Table 4).

#### Experienced observer

With a very high statistical significance (***) biphasic PVS showed the lowest deviation values in the axial and sagittal direction. However, a very strong distribution of values could be detected for the impression material polyether. In the transverse direction it became clear that preliminary PVS exhibited lower deviation values than alginate, polyether and biphasic PVS. This difference was also statistically significant (**). Overall, biphasic PVS stood out with the lowest mean and the lowest standard deviation. Preliminary PVS, alginate and polyether followed with ascending values (Table 5).

**Table 5:** Deviation values for materials by experienced observer in all spatial directions Abweichungswerte für die Materialien bezogen auf den erfahrenen Beobachter in allen räumlichen Ebenen

Orientation	Impression Material	*N*	Mean (mm)	±SD (mm)	*p*-values
Axial	Polyether	48	−0.164	±0.455	0.000 (***)
	Preliminary PVS	48	0.017	±0.127	
	Alginate	48	0.111	±0.220	
	Polyvinylsiloxane	48	0.012	±0.124	
Transversal	Polyether	48	0.040	±0.229	0.001 (**)
	Preliminary PVS	48	−0.005	±0.087	
	Alginate	48	−0.028	±0.092	
	Polyvinylsiloxane	48	−0.061	±0.079	
Sagittal	Polyether	48	−0.242	±0.332	0.000 (***)
	Preliminary PVS	48	−0.124	±0.114	
	Alginate	48	−0.127	±0.120	
	Polyvinylsiloxane	48	0.043	±0.071	
Overall	Polyether	144	0.255	±0.292	0.000 (***)
	Preliminary PVS	144	0.101	±0.084	
	Alginate	144	0.134	±0.123	
	Polyvinylsiloxane	144	0.085	±0.058	

*N* number, *SD* standard deviation, *PVS* polyvinylsiloxane

**p< 0,01, ***p< 0,001

### Differences in the sequence and method of application

Evaluation of the differences regarding the method of application for each impression material revealed that significant differences could only be observed for two materials each in one direction, i.e., in the transverse direction for polyether and in the axial direction for biphasic PVS. While these significances alone were very low (0.049 and 0.046) and therefore would have to be regarded as merely incidental, the difference increased in significance for PVS (0.042), whereas it was lost for polyether (0.117) in overall directions. None of these differences could withstand Bonferroni correction (Table 6).

**Table 6:** Differences in method over material Unterschiede in der Methode gegenüber dem Material

Impression material	Direction	Method of application	*N*	Mean (mm)	±SD (mm)	*p*-values
Polyether	Axial	Local	48	0.037	±0.286	0.251 (n. s.)
		In Tray	48	−0.110	±0.464	
	Transversal	Local	48	0.034	±0.173	0.049 (*)
		In Tray	48	−0.040	±0.215	
	Sagittal	Local	48	−0.179	±0.242	0.080 (n. s.)
		In Tray	48	−0.147	±0.298	
	Overall	Local	144	0.173	±0.193	0.117 (n. s.)
		In Tray	144	0.237	±0.264	
Preliminary PVS	Axial	Local	48	0.101	±0.192	0.087 (n. s.)
		In Tray	48	0.029	±0.153	
	Transversal	Local	48	−0.021	±0.105	0.665 (n. s.)
		In Tray	48	−0.032	±0.094	
	Sagittal	Local	48	−0.133	±0.115	0.684 (n. s.)
		In Tray	48	−0.128	±0.112	
	Overall	Local	144	0.118	±0.089	0.998 (n. s.)
		In Tray	144	0.124	±0.114	
Alginate	Axial	Local	48	0.008	±0.284	0.202 (n. s.)
		In Tray	48	0.071	±0.284	
	Transversal	Local	48	−0.022	±0.124	0.123 (n. s.)
		In Tray	48	−0.055	±0.101	
	Sagittal	Local	48	−0.192	±.240	0.898 (n. s.)
		In Tray	48	−0.158	±0.130	
	Overall	Local	144	0.170	±0.184	0.752 (n. s.)
		In Tray	144	0.156	±0.148	
PVS	Axial	Local	48	0.027	±0.106	0.046 (*)
		In Tray	48	−0.039	±0.158	
	Transversal	Local	48	−0.072	±0.078	0.420 (n. s.)
		In Tray	48	−0.057	±0.095	
	Sagittal	Local	48	−0.016	±0.099	0.881 (n. s.)
		In Tray	48	−0.031	±0.136	
	Overall	Local	144	0.086	±0.060	0.042 (*)
		In Tray	144	0.110	±0.083	

*N* number, *SD* standard deviation, *n.s*. not significant, *PVS* polyvinylsiloxane

*p< 0,05

A univariate ANOVA was not valid due to missing normality with very high standard deviations. Between-subject effects were considered nevertheless, showing low significance for observer (*p* = 0.437) and application method (*p* = 0.177) on the one hand, but higher significance for material (*p* < 0.001) and axis (*p* < 0.001) on the other hand.

### Other relevant factors affecting the choice of impression material

The costs and setting times of all the materials were combined in order to obtain a thorough comparison of the qualities of the impression materials. According to the literature, extending the setting time to 8 min is recommended for polyether because this is associated with improved elastic recovery capacity ([3]; Table 7).

**Table 7:** Comparison of materials concerning costs (in Euro) and setting time (in min) Vergleich der Materialien bezüglich der Kosten (EUR) und der Abbindezeit (min)

	Alginate	Polyether	PVS	PVS preliminary
	Palgat Plus Quick	Impregum Penta refill	Imprint 4 Penta putty + light	Imprint 4 preliminary
Price per impression	1.38	16.46	16.06	6.31
Price relation to alginate	1	11.90	11.61	4.56
Price difference to alginate	0	15.07	14.67	4.92
Setting time (minutes)	2	6–8^1^	6–8 ^2^	2

*PVS *polyvinylsilane

^1^ Increased setting time may improve restitution of distortions

^2^ 2 additional minutes, if materials are used consecutively (correction impression)

## Discussion

The purpose of this study was to ascertain the three-dimensional accuracy of impression materials commonly in use for transfer of the abutment position of an orthodontic miniscrew system (OrthoEasy® Pal, Forestadent Bernhard Förster GmbH, Pforzheim, Germany). The clinical relevance of this study results from the fact that, despite inaccurate impressions of miniscrews on the resulting model, adaptation of the orthodontic appliance is in fact possible [6, 8, 27] but this can prove to be very time-intensive, leading to increased chair time and reduced patient satisfaction.

During polyether impression-taking it was found that, in the experienced observer group, subjectively very strong forces had to be applied to remove the impression from the model. This might have had the impact in this group of a deviation in the measured values caused by relevant deformations in the material. (Comparison of the differences for polyether in the group of experienced versus inexperienced observer.) This high resistance to demoulding is generally recognized in the literature as a complicating factor for polyethers [18]. As no differences that might have had significant effects on the later measurements were observed during pouring, no models were excluded from the study.

In line with the comparable literature regarding the eligibility of impression materials commonly used at present for orthodontic purposes, the results of this study show reasonably low deviation levels for most of the materials [9, 23, 27]. However, an excessive number of outliers for all materials was observed, with the exception of biphasic PVS. While the transverse axis was least affected, the greatest effects appeared in the sagittal and especially the vertical direction.

Based on comparison over all directions, the impression materials can be arranged in order of decreasing accuracy. Thus, biphasic PVS achieved the smallest means of deviation and dispersion, followed by monophasic PVS, alginate and polyether with the weakest performance. This correlates with findings from the current literature which describe PVS in the putty-wash or heavy body-wash combinations as a high-precision standard impression material for prosthodontics and orthodontics, compared with polyether with lower accuracy [2, 10].

Analysis of the differences between the impression materials separately for each observer showed a few interesting subgroup effects. Consideration of the inexperienced observer revealed the lowest deviation values in the vertical direction for biphasic PVS and alginate, whereas polyether and biphasic PVS achieved the lowest values with respect to the sagittal direction. No significant differences were observed in the transverse direction. Regarding all the directions as a whole, the sequence of materials arranged according to increasing mean values was different: polyether achieved a higher level of accuracy than alginate. The most probable explanation for this may lie in the methodological inaccuracies of the inexperienced observer with this standard material.

However, biphasic PVS showed the lowest deviation values for the experienced observer in the vertical and sagittal direction, whereas monophasic PVS stood out in the transverse direction. Arrangement of the materials for all directions as a whole lead to the same result as when both observers are considered together: the material polyether again exhibited very high values. In view of the fact that polyether impressions were the last to be carried out, this might reflect the increased dehydration of the tissues resulting from the continuous impression-taking process.

Regarding the application method, this study reveals a general trend of low mean values and standard deviations for local application, although the significance for this effect was low.

Whereas biphasic PVS in the putty-wash or heavy body-wash technique provided the highest accuracy in casting this orthodontic miniscrew system, the largest deviations and variations in all spatial directions were observed with the material polyether. One possible reason for this may be the tray design with a differing retention effect, as referred to in the study by Steinhäuser-Andresen et al. [23]. On the other hand, there may also have been an increase in undercuts resulting from a reduction of the gingiva and an increase in tooth adherence due to dehydration of the preparation which the polyether material may have been especially susceptible to due to its low elasticity. According to the study by Balkenhol et al., an improvement in elastic recovery capacity was possible by lengthening the setting time to 8 min [3].

Adequate accuracy could be achieved with the alginate impression, which applies particularly to the experienced observer and the procedure in which optimal intraoral accuracy of fit can be achieved by later adaptation [8, 23]. The preliminary polyvinylsiloxane also delivered acceptable results, although temperature was found to have a strong influence on the material for this impression as it disintegrated at a room temperature of 30 °C. In view of the results of this study, monophasic polyvinylsiloxane can be classified as preferable to alginate on the basis of its better dimensional stability in sterilization processes.

According to the current literature, the spatial relation of the screw heads has great importance for stability. An investigation of the accuracy of this relation could be added to this study. On the other hand, general accuracy of the impressions should correlate with the reciprocal accuracy of the screw heads.

For everyday clinical practice, the choice of impression material requires an indication-specific approach with a view to a cost–benefit evaluation and it should be borne in mind that new techniques of intraoral adaptation reduce the need for accuracy [6, 8, 27]. Still, a small deviation at the screw head may, via the lever arm of the device fixed to it, e.g., a transpalatal arch, lead to significant deviations at the tooth level, which is of little consequence with intraoral adaptation but may result in unwanted tooth movement if the prefabricated device cannot be modified intraorally.

Guided scanning methods have become established in orthodontics as well as prosthodontics for diagnostic and therapeutic purposes. The digital impression technique can rule out some of the problems of conventional impression-taking, such as choosing the impression tray, dosing, polymerization and dimensional changes to the impression material, disinfection, and dispatch to the laboratory. Patient comfort is an additional advantage.

It is stated in the current literature that systems of digital impression-taking achieve the same or higher precision levels than some conventional impression materials and, given the correct scanning technique, promise good clinical results [10, 22]. In their systematic review concerning the diagnostic accuracy and sensitivity of the analysis of digital models for orthodontic purposes, Rossini et al. concluded that digital models are as accurate, reliable, and reproducible as classic dental casts [22]. In their study Al Mortadi et al. reported the successful fabrication of an orthodontic appliance in acrylic resin with alloy components using digital technology without a conventional impression [1]. In addition, Graf et al. described the production of an implant-supported appliance for a patient using a CAD-CAM procedure without a physical impression or a printed model [11].

Thus, intraoral three-dimensional impression-taking with its advantages in terms of cost, time, and space required looks promising, especially in the area of digital orthodontic working practices.

## Conclusions

Based on this study, it may be concluded that biphasic polyvinylsiloxane (PVS), for both the experienced and inexperienced observer, is the optimal material for taking impressions for this system of abutment-carrying orthodontic miniscrews. The double mix technique should be regarded as the preferred method of application because of its comparably short setting time of 6 min and high accuracy, which justify the high costs. However, all other impression materials should be considered as second-choice methods because of their high standard deviation values. Provided clinicians are familiar with the concept of intraoral adaptation of palatal appliances, alginate and preliminary PVS can be used for application in view of their short setting time of 2 min and financial considerations. Polyether did not exhibit any favorable results, with high deviations, longest setting time, and strong removal forces. This may be attributed to an increased number of undercuts in the specimen and tray design. Further studies should use optimal trays for each material.
